# Existence detection and embedding rate estimation of blended speech in covert speech communications

**DOI:** 10.1186/s40064-016-2691-6

**Published:** 2016-07-11

**Authors:** Lijuan Li, Yong Gao

**Affiliations:** College of Electronics and Information Engineering, Sichuan University, Chengdu, 610064 Sichuan China

**Keywords:** Blended speech, Covert speech communication, Embedding rate estimation, Existence detection, Odd–even difference (OED)

## Abstract

Covert speech communications may be used by terrorists to commit crimes through Internet. Steganalysis aims to detect secret information in covert communications to prevent crimes. Herein, based on the average zero crossing rate of the odd–even difference (AZCR-OED), a steganalysis algorithm for blended speech is proposed; it can detect the existence and estimate the embedding rate of blended speech. First, the odd–even difference (OED) of the speech signal is calculated and divided into frames. The average zero crossing rate (ZCR) is calculated for each OED frame, and the minimum average ZCR and AZCR-OED of the entire speech signal are extracted as features. Then, a support vector machine classifier is used to determine whether the speech signal is blended. Finally, a voice activity detection algorithm is applied to determine the hidden location of the secret speech and estimate the embedding rate. The results demonstrate that without attack, the detection accuracy can reach 80 % or more when the embedding rate is greater than 10 %, and the estimated embedding rate is similar to the real value. And when some attacks occur, it can also reach relatively high detection accuracy. The algorithm has high performance in terms of accuracy, effectiveness and robustness.

## Background

Covert audio communication refers to the technique of embedding secret information into carrier audio such that important information can be transmitted safely and reliably via public communication. This technology can also be used by terrorists to commit criminal activities, which pose a serious danger to society (Qiao et al. 2013). Traffickers and terrorists have begun to hide secret information in audio files, such as MP3 and WAV files, and spread them by file sharing or e-mail through high-quality Internet (Gelfand 2007).

The speech, as an important branch of the audio, is one of the most important methods for human communication and is ubiquitous and accessible. Currently, existing covert speech communication algorithms (Singh 2016; Tayel et al. 2016; Hartoko et al. 2015; Krishnan and Abdullah 2016; Nutzinger and Juergen 2011; Matsuoka 2006; Byeong-Seob et al. 2005; Tatsuya and Kotaro 2015; Chen 2001) include the following: least significant bits (LSB) hiding (Tayel et al. 2016; Hartoko et al. 2015; Krishnan and Abdullah 2016), phase coding hiding (Nutzinger and Juergen 2011), direct sequence spread spectrum (DSSS) encoding hiding (Matsuoka 2006), echo hiding (Byeong-Seob et al. 2005; Tatsuya and Kotaro 2015), and blending-based speech hiding (Chen 2001), along with others. Among these algorithms, the blending-based speech hiding algorithm is different from others, where the secret speech can be hidden directly in the carrier speech and does not need to be binary encoded. This algorithm has a good robustness (Rangding et al. 2004) and high hidden capacity.

In contrast with covert speech communication, the aim of speech steganalysis (Natarajan and Nayak 2010; Ghasemzadeh et al. 2016; Bhattacharyya and Sanyal 2012; Chunhui and Yimin 2010; Wei et al. 2008; Hamza et al. 2003) is to detect the existence of secret information in covert speech communication and extract that information. Steganalysis algorithms can be divided into two classes based on their scope of application. One class is specific steganalysis algorithms (Chunhui and Yimin 2010; Wei et al. 2008), and the other is universal steganalysis algorithms (Hamza et al. 2003; Özer et al. 2006; Avcıbas 2006). However, no experiment has determined whether existing steganalysis algorithms are applicable to blended speech.

For the above reasons, this paper proposes a steganalysis algorithm for blended speech transmitted via a high-quality Internet. The algorithm is based on the average zero crossing rate (Muhammad 2015; Ali et al. 2011; Ghosal and Suchibrota 2014) of the odd–even difference (AZCR-OED) of the speech, and it combines a support vector machine (SVM) classifier (Mathias and Mohamed 2015; Alex and Bernhard 2004) and a voice activity detection (VAD) algorithm (Shota et al. 2016; Qi et al. 2002; Xinyan and Guojun 2013; Jongseo et al. 1999). The algorithm can detect the existence and estimate the embedding rate of blended speech. The experimental results demonstrate the high accuracy, effectiveness and robustness of this algorithm for a variety of embedding rates.

This paper is organized as follows: “Blending-based speech hiding algorithm” section provides a brief introduction to the blending-based speech hiding algorithm. The features, such as the AZCR-OED of the speech, are analyzed in “Feature analysis” section. A steganalysis algorithm for blended speech, which is used for existence detection and embedding rate estimation, is proposed in “Steganalysis algorithm for blending speech” section. Experimental results and analyses are given in “Experimental results and analysis” section. Finally, “Conclusion” section presents the conclusions of the work.

## Blending-based speech hiding algorithm

*P* denotes the carrier speech of length *N*, and *S* denotes the secret speech of length *M*. The blending-based speech hiding algorithm is described as follows (Nutzinger and Juergen 2011):

1 $$\left\{ {\begin{array}{*{20}l} {F(2k - 1) = P(2k - 1)} \hfill \\ {F(2k) = (1 - \alpha )P(2k - 1) + \alpha S(k)} \hfill \\ \end{array} } \right.\begin{array}{*{20}c} \cdot & {(1 \le k \le M} \\ \end{array} )$$

Considering that the hidden location of secret speech is uncertain, the Eq. (1) can be rewritten as follows:

2 $${\text{First:}}\quad F(k) = P(k),\quad 1 \le k \le N$$

3 $${\text{Then}}{:}\quad \left\{ {\begin{array}{*{20}l} {F(2k - 1 + start) = P(2k - 1 + start)} \hfill \\ {F(2k + start) = (1 - \alpha )P(2k - 1 + start) + \alpha S(k).} \hfill \\ \end{array} } \right.$$

where $1 \le k \le M$, and $0 \le \alpha \le 1$, $\alpha$ is the hidden degree factor; *start* is the location of the start of the secret speech, where $start \in \{ 0,1,2, \ldots ,N - 2M\}$; and *F* denotes the blended speech of length *N*. *F* is more similar to the carrier speech *P* as $\alpha$ decreases. Furthermore, Eq. (3) implies that *M* and *N* must satisfy $N \ge 2M$.

In this paper, we define a stego speech segment as a speech segment in which secret speech is hidden in blended speech. From Eq. (3), we know that the stego segment’s length is $2M$. The embedding rate is defined as the ratio of the length of the stego speech segment to the entire length of the blended speech and is denoted by $\eta$. Thus,

4 $$\eta = \frac{2M}{N} \times 100\,\%$$

where $0 \le \eta \le 100\,\%$, $\eta = 0$ means there is no stego speech segment in the speech signal, and $\eta = 100\,\%$ means the carrier speech is completely used to hide the secret speech. Generally, for a fixed length of secret speech, a lower embedding rate requires a longer carrier speech signal, which leads to lower communication efficiency.

Given Eq. (3), the extraction algorithm for secret speech can be defined as follows:

5 $$\begin{aligned} S(k) & = \frac{F(2k + start) - (1 - \alpha )P(2k - 1 + start)}{\alpha } \\ & = \frac{F(2k + start) - (1 - \alpha )F(2k - 1 + start)}{\alpha } \\ \end{aligned}$$

where $1 \le k \le M$. From Eq. (5), we can observe that if the receiver wants to extract the secret speech from the blended speech, he/she must know at least the following information:

1. the location of the start of the secret speech;
2. whether the odd–even points are aligned (which is defined below) with the sender;
3. the hidden degree factor $\alpha$ of the secret speech.

The aims of this paper are as follows: extract the features that can distinguish pure speech from blended speech to detect blended speech, judge whether the odd–even points are aligned with the sender and correct the inverted case, estimate the hidden starting location of the secret speech and the length of the stego speech segment, and calculate the embedding rate.

## Feature analysis

In this section, we first briefly state several definitions that will be used later. Then, the difference in the odd–even difference (OED) between the blended speech and pure carrier speech is analyzed and compared. Finally, we present the features that can distinguish pure and blended speech.

### Definitions

#### **Definition 1**

For the speech signal *X*($X = \{ x(1),x(2),x(3), \ldots ,x(N)\}$), the OED is defined to be the difference between the values of odd and even points. Denoting the OED by *D*, we have $D(k) = x(2k) - x(2k - 1)$, where $1 \le k \le \left\lfloor {N/2} \right\rfloor$ and $\left\lfloor \cdot \right\rfloor$ denotes the rounded-down value.

#### **Definition 2**

For a sent speech signal *X*($X = \{ x(1),x(2),x(3), \ldots ,x(N)\}$) and received speech signal *R*($R = \{ r(1),r(2),r(3), \ldots ,r(M)\}$), when $r(n) = x(n)$ (where $1 \le n \le N$), we consider the odd–even points of the received speech to be aligned with the sent speech. When $r(2k - 1) = x(2k)$ and $r(2k) = x(2k + 1)$, where $1 \le k \le \left\lfloor {N/2} \right\rfloor$, we define the odd–even points of the received speech to be inverted with respect to the sent speech.

#### **Definition 3**

For the speech signal *X*($X = \{ x(1),x(2),x(3), \ldots ,x(N)\}$), the average zero crossing rate (ZCR) (Muhammad 2015; Ali et al. 2011; Ghosal and Suchibrota 2014) is defined as follows:

$$Z = \frac{1}{2N}\sum\limits_{k = 2}^{N} {\left| {\text{sgn} [x(k)] - \text{sgn} [x(k - 1)]} \right|} ,$$

where $$\text{sgn} [x(k)] = \left\{ {\begin{array}{*{20}l} {1,} \hfill &\quad {x(k) \ge 0} \hfill \\ { - 1,} \hfill &\quad {x(k) < 0} \hfill \\ \end{array} } \right.$$.

### OED of the speech signal

*X* and *R* denote the pure sent speech signal and the received speech signal, respectively. Four cases of the OED of received speech are discussed below.

It is assumed that the hidden starting location of the secret speech is $start = 0$ and that the embedding rate is $\eta = 100\,\%$. For other values, the derivation is similar.

Case 1: the received speech is the blended speech, and the odd–even points are aligned with the sent speech. The OED denoted by $Drc$ is as follows:

6 $$\begin{aligned} Drc(k) & = R(2k) - R(2k - 1) \\ & = F(2k) - F(2k - 1) \\ & = (1 - \alpha )P(2k - 1) + \alpha S(k) - P(2k - 1) \\ & = \alpha [S(k) - P(2k - 1)]. \\ \end{aligned}$$

Case 2: the received speech is the pure speech, and the odd–even points are aligned with the sent speech. The OED denoted by $Dro$ is as follows:

7 $$Dro(k) = R(2k) - R(2k - 1) = X(2k) - X(2k - 1).$$

Case 3: the received speech is the blended speech, and the odd–even points are inverted with respect to the sent speech. The OED denoted by $Dwc$ is as follows:

8 $$\begin{aligned} Dwc(k) & = R(2k) - R(2k - 1) \\ & = F(2k + 1) - F(2k) \\ & = P(2k + 1) - (1 - \alpha )P(2k - 1) + \alpha S(k). \\ \end{aligned}$$

Case 4: the received speech is the pure speech, and the odd–even points are inverted with respect to the sent speech. The OED denoted by $Dwo$ is as follows:

9 $$Dwo(k) = R(2k) - R(2k - 1) = X(2k + 1) - X(2k).$$

**Fig. 1:**
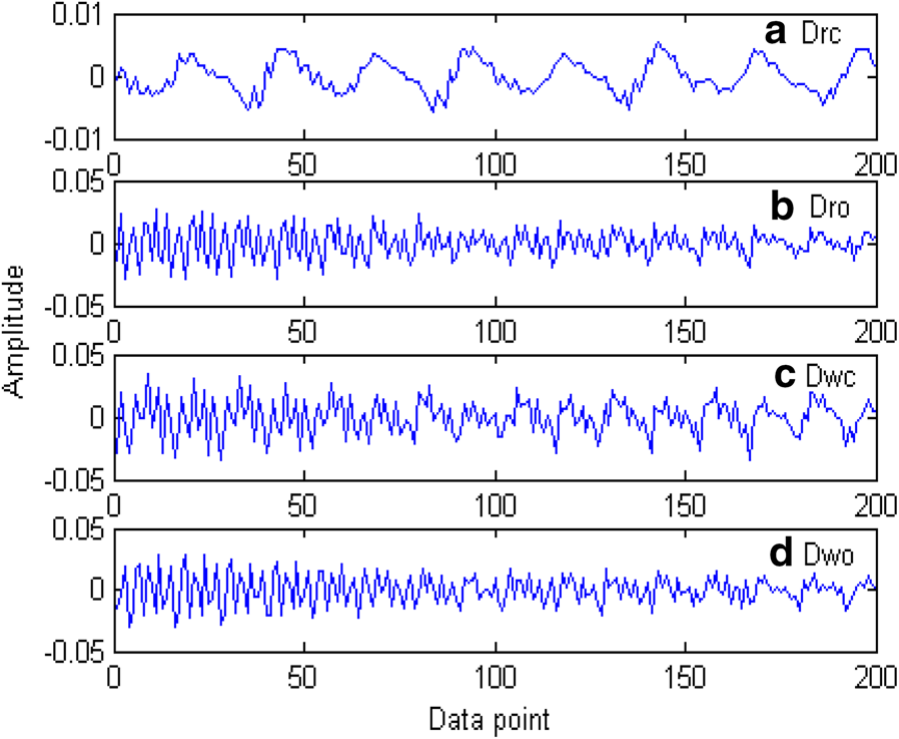
OEDs of the pure speech and the blended speech: **a** $Drc$, **b** $Dro$, **c** $Dwc$, and **d** $Dwo$

We selected two pure speech samples from the Voice of America (VOA) Special English corpus (http://www.51voa.com/VOA_Special_English/) randomly and transformed them into the WAV format with an 8-kHz sampling rate and 16-bit speech encoding. Then, one of the samples was chosen to be the secret speech and hidden in the other using the blending-based speech hiding algorithm, with an embedding rate of 100 % and a hidden degree factor $\alpha = 0.05$. Finally, both the pure and blended speech were transmitted through QQ, a popular instant messaging software service in China that is used for chatting, and the receiver analyzed the OED of the received speech. The result is shown in Fig. 1 (to see the result clearly, only data points 1–200 are shown in the figure).

We can intuitively see from Fig. 1 that the change in the OED of the blended speech when the odd–even points are aligned with the sent speech, which is denoted by $Drc$, is less rapid than in the other three cases. In this paper, we use the average ZCR to describe this difference.

### AZCR-ODE of the speech signal

According to definition 3 and Eq. (6), the corresponding AZCR-OED of $Drc$ can be determined as follows:

10 $$\begin{aligned} Z(Drc) & = \frac{1}{2N}\sum\limits_{k = 2}^{N} {\left| {\text{sgn} [Drc(k)] - \text{sgn} [Drc(k - 1)]} \right|} \\ & = \frac{1}{2N}\sum\limits_{k = 2}^{N} {\left| {\text{sgn} \{ \alpha [S(k) - P(2k - 1)]\} - \text{sgn} \{ \alpha [S(k - 1) - P(2k - 3)]\} } \right|} \\ & = \frac{1}{2N}\sum\limits_{k = 2}^{N} {\left| {\text{sgn} [S(k) - P(2k - 1)] - \text{sgn} [S(k - 1) - P(2k - 3)]} \right|} . \\ \end{aligned}$$

In Eq. (10), we can see that $Z(Drc)$ is determined only by the value of the secret speech and the carrier speech and is unrelated to the hidden degree factor of the secret speech.

To verify whether the AZCR-OED can serve as feature to distinguish between blended and pure speech, we first obtained 8000 pure speech samples from the VOA Special English corpus (http://www.51voa.com/VOA_Special_English/.). Then, we transformed all of them into the WAV format with an 8-kHz sampling rate and 16-bit speech encoding and built a speech sample library (which is hereafter referred to as the “VOASE” library). Finally, we divided the VOASE library into two groups, the secret speech group and the carrier speech group. We performed a statistical analysis of the AZCR-OED of the blended speech and pure speech through the following experiments.

**Fig. 2:**
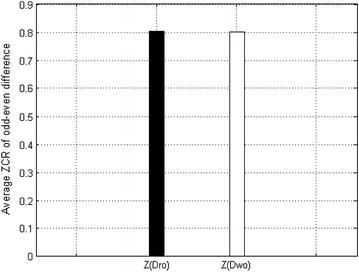
Statistical results for $Z(Dro)$ and $Z(Dwo)$, the AZCR-OED of pure speech when the odd–even points are aligned and inverted, respectively

Experiment 1: we calculated the OEDs $Dro$ and $Dwo$ for the pure speech in the VOASE library under both conditions, where the odd–even points are aligned and inverted, along with the corresponding average ZCR values, $Z(Dro)$ and $Z(Dwo)$. Figure 2 shows the statistical results.

**Fig. 3:**
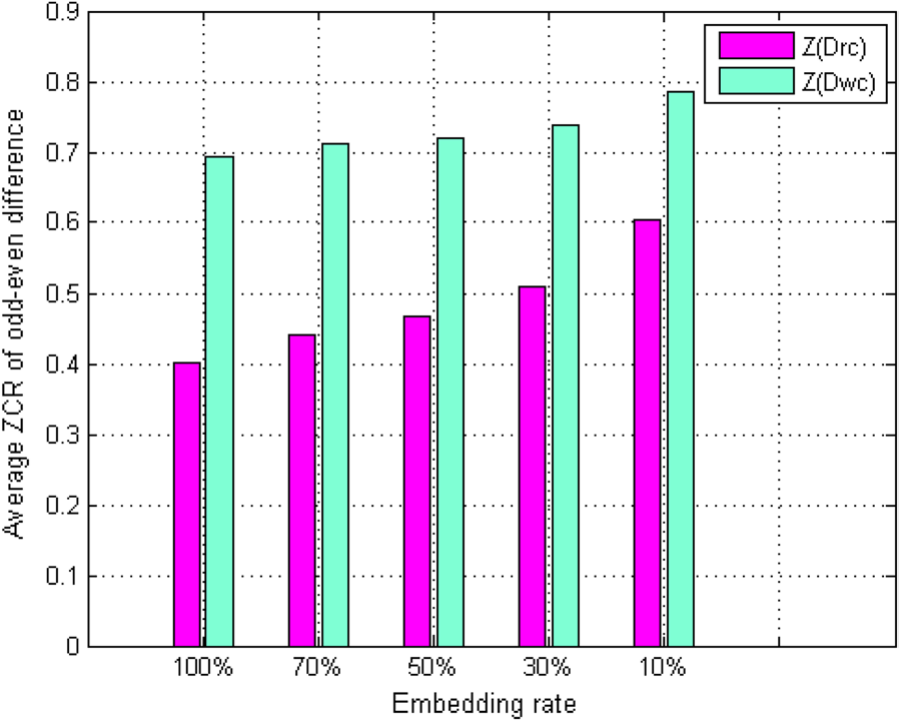
Statistical results for $Z(Drc)$ and $Z(Dwc)$, the AZCR-OED of blended speech when the odd–even points are aligned and inverted, respectively

Experiment 2: we made five copies of the carrier speech group. Then, secret speech was embedded into the carrier speech signal using the blending-based speech hiding algorithm with five different embedding rates. Because the embedding rate is typically high in practical applications, we selected 10, 30, 50, 70, and 100 % as the embedding rates in the experiment. When the odd–even points are aligned, the AZCR-OED of blended speech is unrelated to the hidden degree factor. Thus, we used a hidden degree factor of 0.1 in the experiment. Consequently, we obtained five blended speech groups with different embedding rates. We calculated the OED $Drc$ of each blended speech signal and the corresponding average ZCR $Z(Drc)$. Then, we inverted the odd–even points of each blended speech, and calculated the OED $Dwc$ of each inverted blended speech signal as well as the corresponding average ZCR $Z(Dwc)$. Figure 3 shows the statistical results.

**Fig. 4:**
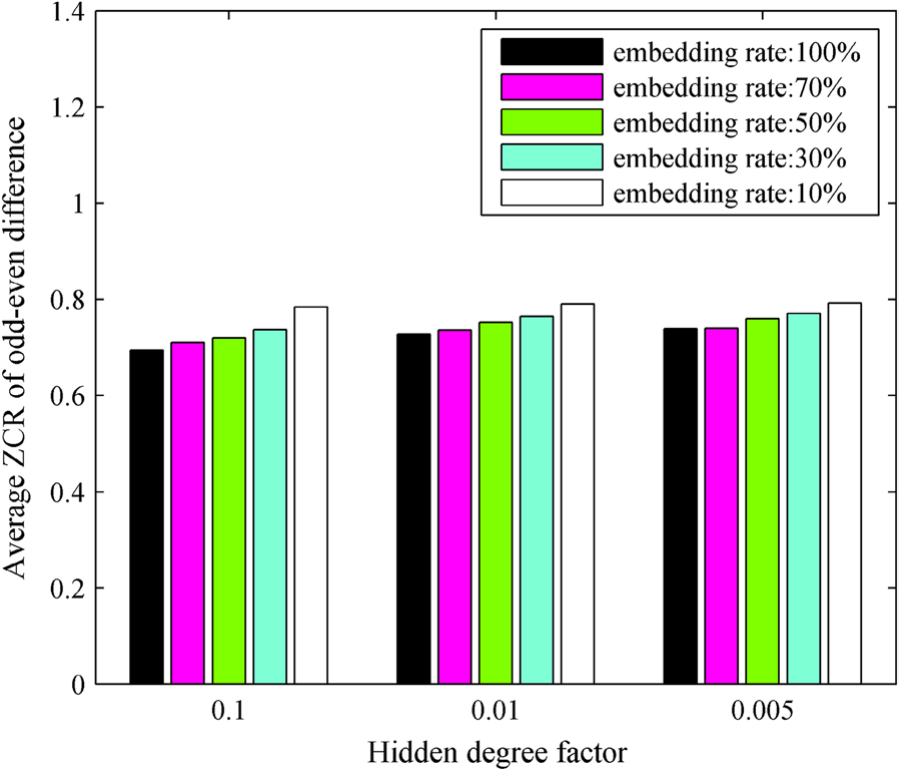
Statistical results for $Z(Dwc)$, the AZCR-OED of blended speech when the odd–even points are inverted

Experiment 3: we made fifteen copies of the carrier speech group and then embedded secret speech into the carrier speech using the blending-based speech hiding algorithm with fifteen combinations of three hidden degree factors and five embedding rates. In the experiment, the hidden degree factors were 0.1, 0.01, and 0.005, and the embedding rates were 10, 30, 50, 70, and 100 %, respectively. Thus, we obtained fifteen blended speech groups. We inverted the odd–even points of the speech in the blended speech groups and calculated the OED $Dwc$ of each inverted blended speech signal and the corresponding average ZCR $Z(Dwc)$. Figure 4 shows the statistical results.

From Fig. 2, we can observe that whether the odd–even points are aligned has almost no effect on the AZCR-OED of the pure speech, i.e.,$Z(Dro) \approx Z(Dwo)$.

From Fig. 3, we can observe that $Z(Drc)$ and $Z(Dwc)$, which are the AZCR-OED of the blended speech when the odd–even points are aligned and inverted respectively, increase as the embedding rate decreases. Comparing $Z(Drc)$ and $Z(Dwc)$, we can see that the former is significantly less than the latter when the embedding rate is the same.

From Fig. 4, we can observe that the hidden degree factor of the secret speech has little effect on $Z(Dwc)$ when the embedding rate is kept fixed.

Comparing Figs. 2 and 3, we can see that $Z(Drc)$ is significantly less than $Z(Dro)$ and $Z(Dwo)$ when the embedding rates are different.

From the experimental results, we can conclude that it is feasible and effective to use the AZCR-OED of the speech as a feature to distinguish between blended and pure speech.

## Steganalysis algorithm for blending speech

In this section, we use the differences of the AZCR-OED between blended speech and pure speech to build a steganalysis algorithm for blended speech. The algorithm can achieve the following under a variety of embedding rates.

1. It can detect the existence of secret speech.
2. For blended speech, it can determine the hidden location of the secret speech and estimate the embedding rate.

### Existence detection of blended speech

Because the AZCR-OED of blended and that of pure speech have obvious differences, when the odd–even points of the blended speech are aligned, the AZCR-OED is lower than when they are inverted. In this paper, according to the features of the blended speech, we first correct the case in which the odd–even points are inverted and then extract the features and use a SVM classifier to detect the existence of blended speech. Table 1 summarizes the procedure for extracting the features from a speech signal.

**Table 1:** Algorithm for feature extraction

**Input**: A speech signal *X* of length *N*
**Output:** A feature vector *F* that contains two features
*Step 1*: For a given speech signal *X*, invert its odd–even points to obtain the inverted version $X_{w}$
*Step 2*: Calculate the OED of *X*, which is denoted by $D_{r}$, and the OED of $X_{w}$, which is denoted by $D_{w}$
*Step 3*: Calculate the average ZCRs of $D_{r}$ and $D_{w}$, respectively, which are denoted by $Z(D_{r} )$ and $Z(D_{w} )$. Obtain the first feature $v_{1}$, where $v_{1} = \hbox{min} \{ Z(D_{r} ),Z(D_{w} )\}$. Here, the reason we take the smaller average ZCR is to correct the case in which the odd–even points of the blended speech are inverted
*Step 4*: If $Z(D_{r} ) \le Z(D_{w} )$, set $D = D_{r}$; otherwise, $D = D_{w}$. Divide *D* into *N* frames and calculate the average ZCR per frame, choosing the smallest value as the second feature, which is denoted by $v_{2}$
*Step 5*: Construct the feature vector $F = < v_{1} ,v_{2} ,Type >$ from these two features. The type attribute specifies whether the signal is blended (1) or pure (−1) speech object

Comparing Fig. 2 with Fig. 3, we see that the differences in the AZCR-OED between blended and pure speech are reduced when the embedding rate is low. It is thus easy to make a misjudgment using the SVM classifier. However, when the AZCR-OED of the stego speech segments is low, calculating the average ZCR per frame and extracting the minimum one as the feature can enhance the differences between blended speech and pure speech, and misjudgment can be reduced. For this reason, the second feature $v_{2}$ is chosen.

In this paper, we use the above algorithm to extract the feature vector of the blended and pure speech and then use the freely available software package LIBSVM for training to establish a classifier, finally achieving existence detection of the blended speech.

### Estimation of the embedding rate

For the blended speech, the AZCR-OED of the stego speech segments is less than that of the pure speech segments. Table 2 presents the algorithm to detect the hidden location of the secret speech and estimate the embedding rate.

**Table 2:** Algorithm for estimating the embedding rate

**Input**: A blended speech signal *X* of length *N*
**Output:** Hidden location of the secret speech and the estimated embedding rate
*Step 1*: For a given speech signal *X*, invert its odd–even points to obtain the inverted version $X_{w}$
*Step 2*: Calculate the OED of *X*, which is denoted by $D_{r}$, and the OED of $X_{w}$, which is denoted by $D_{w}$
*Step 3*: Calculate the average ZCRs of $D_{r}$ and $D_{w}$, respectively, which are denoted by $Z(D_{r} )$ and $Z(D_{w} )$, and set $Q_{mean} = \hbox{min} (Z(D_{r} ),Z(D_{w} ))$
*Step 4*: If $Z(D_{r} ) \le Z(D_{w} )$, set $D = D_{r}$; otherwise, $D = D_{w}$
*Step 5*: Divide *D* into *N* frames and calculate the average ZCR per frame, which is denoted by $Q(i)$, where *i* denotes the *i*th frame
*Step 6*: Let $Flag(i)$ denote the symbol of the *i*th frame: $Flag(i) \in \{ 0,1\}$. If $Q(i) < Q_{mean}$, set $Flag(i) = 0$; otherwise, $Flag(i) = 1$
*Step 7*: The hang-over scheme (Avcıbas 2006; Muhammad 2015), which is a type of VAD algorithm, is used for $Flag(i)$ to mark the secret speech segments, thereby ensuring that the hidden location of the secret speech is determined
*Step 8*: Calculate the length of the secret speech segments and the embedding rate

**Fig. 5:**
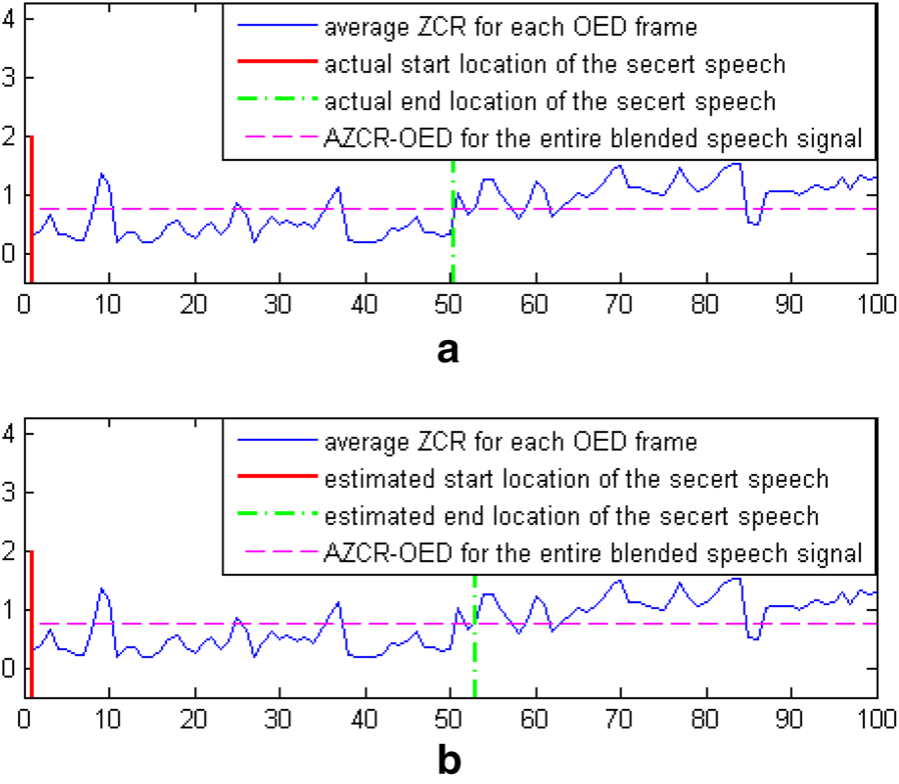
Hidden location of the secret speech: **a** Actual hidden location and **b** Estimated hidden location

We chose two speech samples randomly from the VOASE library. Then, we chose one to be the secret speech and hid it in the other using the blending-based speech hiding algorithm with an embedding rate of 50 % and a hidden degree factor $\alpha = 0.01$. Then, the algorithm presented above was used to detect the hidden location of the secret speech. A frame length of 256 was used. The result is shown in Fig. 5.

From Fig. 5a, we can observe that the AZCR-OED values of most of the stego speech frames are less than the value for the entire blended speech signal, and the AZCR-OED values of most of the pure speech frames are greater than the value for the entire blended speech signal. Comparing panels (a) and (b) of Fig. 5, we see that the estimated hidden location of the secret speech is similar to its actual hidden location. These experimental results demonstrate the effectiveness of the algorithm.

## Experimental results and analysis

In this section, we evaluate the performance of the algorithms for feature extraction and estimation of the embedding rate.

One thousand speech files were randomly selected from the VOASE library and divided into two groups. One group was the secret speech group, which contained 500 secret speech files. Ten copies of the other 500 speech files were made to be used as carrier speech files, which were embedded with secret speech using the blending-based speech hiding algorithm with 10 different embedding rates of 10, 20, 30… 100 %. Because the AZCR-OED of blended speech is unrelated to the hidden degree factor of the secret speech, the hidden degree factor was chosen to be 0.01 in the experiments. Therefore, 500 blended speech signals were obtained for each embedding rate.

### Basic experiments

#### Existence detection of the blended speech

**Fig. 6:**
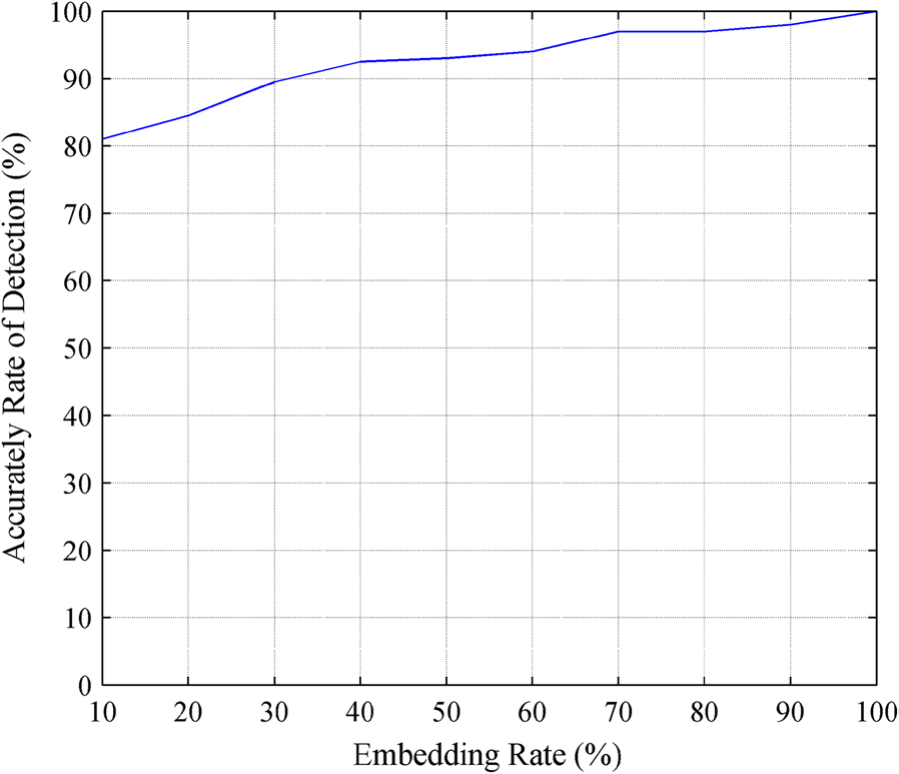
Detection accuracy for ten different embedding rates

We randomly chose 250 blended speech files and 250 pure carrier speech files for each embedding rate and marked their type as 1 or −1, respectively. We then extracted the feature parameters for training the SVM classifier and used the remaining 500 speech samples, including 250 blended and 250 pure speech samples, for testing. Figure 6 shows the experimental results.

From Fig. 6, we can observe that the detection accuracy can be greater than 80 % when the embedding rate is greater than 10 %, and the accuracy of detection increases as the embedding rate increases.

From the results, we conclude that the reason that the detection accuracy decreases as the embedding rate decreases is as follows. The amount of secret speech decreases when the embedding rate decreases, thus, the differences in the AZCR-OED between blended and pure speech decrease. It is therefore easy for the SVM classifier to make a misjudgment, thereby decreasing the detection accuracy. However, for a fixed length of secret speech, a lower embedding rate requires a longer carrier speech signal, which leads to low communication efficiency. Thus, a low embedding rate is not adopted in practical applications of covert speech communication.

#### Embedding rate estimation

Under each actual embedding rate, we used 500 blended speech files to estimate the embedding rate using the algorithm described in “Estimation of the embedding rate” section and then calculated the mean and variance of the estimated embedding rate. The experimental results are presented in Table 3.

**Table 3:** Embedding rate estimation

Actual embedding rate (%)	Estimated embedding rate	
	Mean (%)	Variance
10	12.54	2.80 × 10^−2^
20	21.48	2.50 × 10^−2^
30	30.13	1.98 × 10^−2^
40	40.53	1.79 × 10^−2^
50	51.31	1.79 × 10^−2^
60	60.23	9.2 × 10^−3^
70	69.63	8.1 × 10^−3^
80	81.53	5.3 × 10^−3^
90	90.43	6.7 × 10^−3^
100	98.43	1.64 × 10^−2^

From Table 3, we can observe that the estimated embedding rate is similar to the real value, and the variance is small. The AZCR-OED of the blended speech increases as the embedding rate decreases, which increases the threshold that distinguishes the stego and pure speech frames. Therefore, many pure speech frames were misjudged to be stego speech frames, which caused the embedding rate to be overestimated.

### Experiments for robustness

To testify the robustness of this algorithm, we designed a group of attack experiments on the test speech for each embedding rate, including: (1) Resample: the speech is sampled up to 16-kHz then sampled down to 8-kHz. (2) Requantization: 16-bit encoding speech is converted to 8-bit encoding. (3) Gaussian white noise: the white noise is added with the SNR being 25 dB. (4) G.729 compression encoding.

**Fig. 7:**
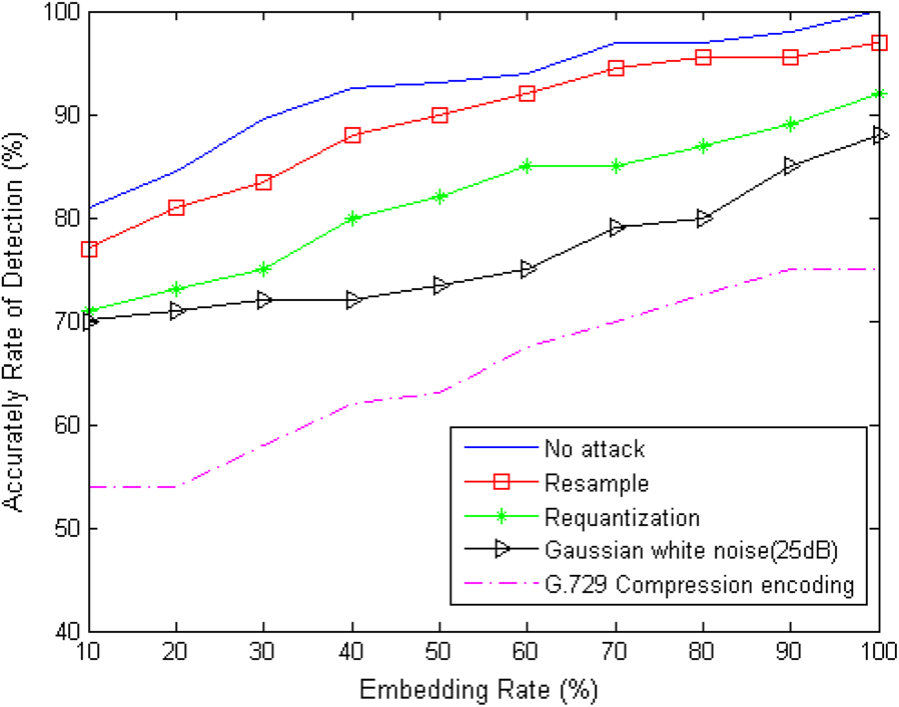
Detection accuracy under different types of attacks

We extracted the feature parameters from the attacked training speeches, and sent them to trained SVM classifier respectively. Then we can detect the existence of secret speech. Figure 7 shows the experimental results (for a better comparison, we redraw the curve in Fig. 7).

From Fig. 7, we can observe that:

1. for the different embedding rate, the detection accuracy without attack is higher than that with attacks;
2. the attacks of resample and requantization have a lower effect on detection accuracy;
3. the attack of Gaussian white noise has a higher effect on detection accuracy;
4. after the speech is encoded with the G.729 compression encoding, the performance of the detection is reduced substantially;
5. under different types of attacks, the detection accuracy increases as the embedding rate increases. Therefore, we can divide a long speech into short speech segments so that we can improve embedding rate in some speech segments of blended speech. Thus, the detection accuracy can be increased.

Similarly, under each actual embedding rate, we processed 500 blended speeches using the aforementioned attacks, and then estimated the embedding rate using the algorithm described in “Estimation of the embedding rate” section. The experimental results are presented in Table 4.

**Table 4:** Embedding rate estimation under different types of attacks

Actual embedding rate (%)	No-attack (%)	The types of attacks			
		Resample (%)	Requantization (%)	Gaussian white noise (25 dB) (%)	G.729 compression encoding (%)
10	12.54	6.87	7.00	12.31	14.07
20	21.48	15.53	16.71	22.32	14.97
30	30.13	24.68	34.06	33.31	22.58
40	40.53	46.01	44.19	43.29	48.36
50	51.31	56.68	54.79	52.61	58.48
60	60.23	65.89	65.18	57.41	67.31
70	69.63	74.52	73.63	67.15	78.12
80	81.53	82.91	83.00	77.78	86.79
90	90.43	87.88	88.35	85.87	82.98
100	98.43	96.64	96.08	94.96	92.21

From Table 4, we can find that no matter what kind of attack it is, there is certain influence on estimated embedding rate. And the attacks lead to a bigger estimated error than the case of no attack. But the estimated embedding rate is around the actual embedding rate.

In conclusion, for the steganalysis algorithm which is proposed in this paper, we can safely conclude that it has a good robustness for the attacks such as resample, requantization. But it is sensitive to Gaussian white noise and G.729 compression encoding.

From the Eq. (3), it can be seen that the algorithm hides the secret speech by modifying the values of even points of carrier speech in the time domain. When Gaussian white noise is imposed on blended speech, the OEDs of the blended speech will be changed significantly due to the randomness of the noise. Especially when the positive or negative prescriptions of the OEDs which are close to zero are changed, it will have large impact on ZCRs of each frame and the entire speech. The probability of misjudgment and the estimated error of embedding rate will then be increased.

G.729 compression encoding divides the speech signal into frames in length of 10 ms. Each encoded signal frame is represented with 80 bits. For the WAV format speech with an 8-kHz sampling rate and 16-bit quantization, the data rate is 128kbps. With a compression rate of 16:1, the data rate will be lowered to 8kbps after the G.729 compression encoding. Consequently, G.729 compression encoding will cause a large loss of numerical information in the time domain of the blended speech. It inevitably leads to that the AZCR-OED is not compliance with the original features any more. Therefore this case also can increase the probability of the misjudgment, as well as the estimated error of embedding rate.

Although the robustness of this steganalysis algorithm is not ideal when speech is attacked by Gaussian white noise or G.729 compression encoding. But in the practical application, the probability is low for the blended speech to be attacked by strong white noise or compression encoding. There are two main reasons. First, the Internet channel has a high-quality. Second, the hidden capacity of the blending-based speech hiding algorithm is high, which results that the blended speech has a low compressibility. In order to extract the secret speech correctly, for both sides of the covert communication, it is less likely to transmit the compression blended speech.

## Conclusion

In this paper, we first briefly introduced the background and significance of this paper along with the blending-based speech hiding algorithm. Then, considering a high-quality Internet, we analyzed the differences in the OED values of blended and pure speech and quantified these differences using the average ZCR. The experimental results verified the correctness of the theoretical analysis. Finally, we proposed a steganalysis algorithm for blended speech. The algorithm successfully achieved existence detection of blended speech and embedding rate estimation under many embedding rates, and the experimental results demonstrate the high accuracy, effectiveness and robustness of this algorithm. Determining how to estimate the hidden degree factor $\alpha$ of secret speech and how to extract the secret speech signal are our future research goals.
